# UMEPPI: An ultrasensitive detection method for protein–protein interaction

**DOI:** 10.17912/micropub.biology.000309

**Published:** 2020-09-14

**Authors:** Mikiya Umeyama, Jun Hirose, Kengo Morohashi

**Affiliations:** 1 Faculty of Science and Technology, Department of Applied Biological Science, Tokyo University of Science, 2641 Yamazaki, Noda, Chiba 278-8510, Japan

**Figure 1:**
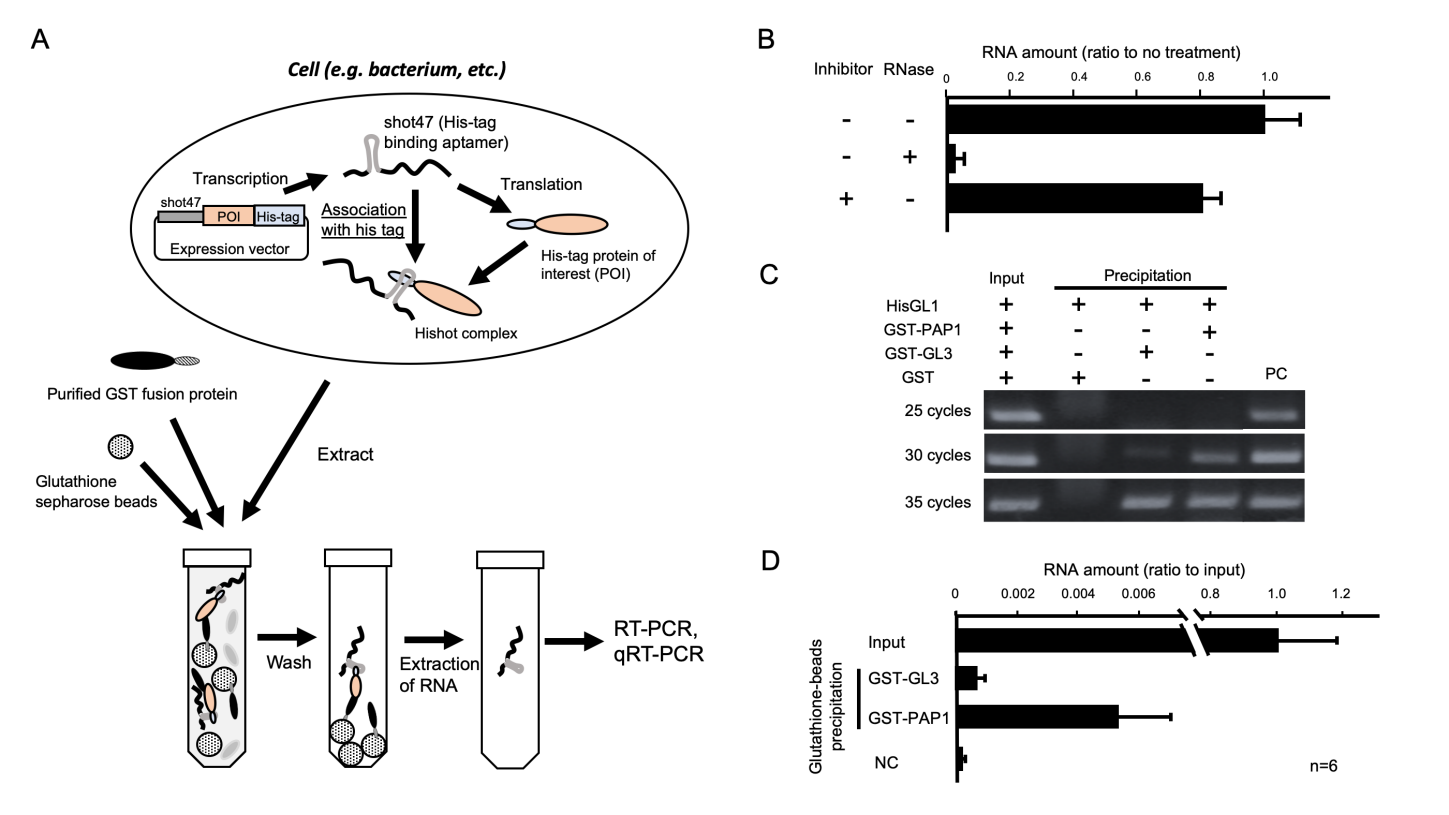
An ultrasensitive detection method for protein–protein interaction. (A) A schematic representation of the UMEPPI method. Shot47 is a ribonucleic acid (RNA) aptamer that strongly associates with a histidine tag. The shot47 complex and protein-of-interest is called the Hishot complex. The interaction can be detected by performing reverse transcription polymerase chain reaction (RT-PCR) or quantitative RT-PCR (qRT-PCR) of purified mRNA of the Hishot complex. (B) The stability of mRNA in the Hishot complex. The Hishot complex was extracted from the expression of the host *E*. *coli* with a presence or absence of RNase I or RNase inhibitor. The mRNA concentration of the Hishot complex is shown as the relative amount compared with the nontreatment. (C) Semi-qRT-PCR of *GL1*. The size of the specifically amplified product from *GL1* CDS is approximately 270 bp. PC indicates a specific *GL1* fragment amplified from the pSpET-GL1 vector. RNA was extracted from precipitates with GST, GST-GL3, or GST-PAP1. (D) The qRT-PCR results. The amount of the *GL1* transcript was shown as a relative amount to the input fraction. The amount of the *GL1* transcript from the Hishot complex co-precipitated with GST-PAP1 and is higher than that with GST-GL3. NC shows a signal from no template fraction. Error bars represent standard deviations.

## Description

The quantification of protein and detection of protein–protein interaction (PPI) largely requires antibody-mediated techniques such as Western blotting and immunoprecipitation. These detection methods for PPI have relatively low sensitivity compared with those for nucleic acids. Because the polymerase chain reaction (PCR) has a powerful amplification of signals derived from nucleic acids, this method for detecting nucleic acids has a higher sensitivity than general PPI detection methods. In this study, we developed a novel method for detecting PPI by converting protein signals to those of nucleic acids, which is called the ultrasensitive detection method for PPI (UMEPPI). UMEPPI uses an RNA aptamer that tightly associates with a histidine tag. The RNA aptamer is genetically fused to a gene encoding a histidine-tagged protein-of-interest (POI). Because the affinity between the RNA aptamer and the histidine peptide tag is pico mol/l (10^−12^ M) order as a dissociation constant (*K_D_*) (Tsuji *et al.*, 2009), the RNA aptamer-tagged protein complex is stable through protein purification steps, and the mRNA of POI can be detected by reverse transcription PCR (RT-PCR). The goal of this study was to prove the concept of UMEPPI. The scheme of UMEPPI is shown in Figure 1A. UMEPPI uses an RNA aptamer called shot47 (Tsuji *et al.*, 2009), which is located upstream of a coding region of the POI with a histidine peptide tag so that mRNA is tightly associated with its own protein through the histidine peptide tag. Thus, the detection of mRNA indicates the existence of shot47 and histidine-peptide-tagged POI, which is called the Hishot complex. If there is a protein interaction with the Hishot complex, the interaction can be detected through the Hishot complex mRNA by RT-PCR. It is important to evaluate the stability of the Hishot complex through the protein extraction step to detect the PPI by UMEPPI. Particularly during the extraction process, endogenous RNase in bacteria or cells may degrade RNA aptamers of the Hishot complex. To investigate the stability of the Hishot complex mRNA, we performed protein extraction with or without RNase and RNase inhibitor treatments followed by detection of the Hishot complex mRNA with quantitative RT-PCR (qRT-PCR). Figure 1B shows that the Hishot complex mRNA was stable even through the bacterial extraction steps, whereas an extra amount of RNase significantly degraded the Hishot complex mRNA. Moreover, the RNase inhibitor did not dramatically change the amount of Hishot mRNA. This suggests that the Hishot complex mRNA showed a minimal effect on endogenous RNase.

For the evaluation of UMEPPI, we used a known combination of PPI that we previously reported (Umeyama and Morohashi, 2020). GLABROUS1 (GL1; At3g27920), PRODUCTION OF ANTHOCYANIN PIGMENT 1 (PAP1; At1g56650), and GLABRA3 (GL3; At5g41315) are transcription factors involved in organ development and metabolite biosynthesis in seed plant, *Arabidopsis thaliana*. Studies previously determined the *K_D_* of GL1 vs. PAP1 and GL1 vs. GL3 are 4.87 × 10^−8^ M and 9.53 × 10^−6^ M, respectively. Because the affinities of GL1 vs. PAP1 and GL1 vs. GL3 show a difference of approximately 190 times, this is a reasonable evaluation system for UMEPPI. Even though any tagged system can be used, we used the glutathione S-transferase (GST) protein system to handle purification and precipitation. GST proteins were fused to the N-termini of PAP1 and GL3 proteins. The GST-PAP1 and GST-GL3 proteins were expressed in bacteria and purified. To create the Hishot complex, we constructed the pSpET vector, in which the coding sequence of GL1 was inserted downstream of the histidine tag and shot47. The crude soluble fraction of the GL1 Hishot complex was incubated with purified GST, GST-GL3, or GST-PAP1 and followed by precipitation using glutathione sepharose beads. We validated whether the GL1 Hishot complex was co-precipitated with GST fusion proteins using RT-PCR. RT-PCR was performed using primer sets designed from part of a coding region of *GL1*. As shown in figure 1C, the extract from the GST-GL3 and GST-PAP1 precipitated fraction clearly shows a signal of *GL1* transcripts, whereas no signal was detected for the GST protein, which suggests that the GL1 Hishot complex is stably precipitated with the GST-GL3 or GST-PAP1 protein. Semi-qRT-PCR suggests that GST-PAP1 would show a higher affinity with the GL1 Hishot complex. We also performed qRT-PCR. Similarly, figure 1D shows that the amounts of co-precipitated mRNA with GST-PAP1 were more than those with GST-GL3. Considering the *K_D_* between 1,450 monoclonal antibody and antigen values in the 1 × 10^−9^ to 2 × 10^−8 ^M range (Landry *et al.*, 2014), the affinity between shot47 and the histidine peptide tag is significantly high (*K_D_* = 3.78 × 10^−12 ^M) (Tsuji *et al.*, 2009), indicating that UMEPPI shows a higher sensitivity than general antibody–antigen approaches. Moreover, UMEPPI successfully detected the difference in these affinities (Figure. 1C and D).

There are various detection methods of PPI that do not require antibodies (e.g., surface plasmon resonance, quartz crystal microbalance, mass spectrometry, the phage display system, and the yeast two hybrid system) (Braun and Gingras, 2012; Rao *et al.*, 2014); however, these approaches are costly and/or labor intensive. Therefore, the antibody-mediated methods are largely used in the field of molecular biology. Nevertheless, antibody-mediated methods have shortcomings.The specificity of the antibody is often variable or unclear. UMEPPI overcomes the problem by using a specific sequence of POI, and the design of the primer sets is straightforward and cost-effective; thus, UMEPPI is a potential and versatile substitute for canonical immunoprecipitation followed by Western analysis. UMEPPI uses PCR as a detection approach and it easily amplifies even very low amounts of protein interactions quantitatively. Ultimately, UMEPPI could become a protein-to-nucleic-acid converter for any type of protein.

## Methods

Construction of the pSpET vector

A total of 50 µM of oligo DNA fragments, UM_5′Xba1_shot47 (5′-CTAGAGGTATATTGGCGCCTTCGTGGAATGTCAGTGCCT-3′) and UM_3′Xba1_shot47 (5′-CTAGAGGCACTGACATTCCACGAAGGCGCCAATATACCT-3′), were mixed in 10 µL of TE (10 mM Tric-HCl pH8.0 and 1 mM EDTA pH8.0). Then, the oligo fragments were heated at 95°C using a thermal cycler. After 5 min, the thermal cycler was turned off, and the fragments were left overnight to reach room temperature. The pET21 vector was treated with XbaI restriction enzyme at 37°C for 1 h. After ethanol precipitation, the digested pET21 and shot47 dsDNA fragments were ligated using a DNA ligation kit <Mighty mix> (TAKARA Bio, Shiga, Japan) at 16°C for 30 min.

Expression and purification of the Hishot complex

The coding sequence (CDS) of GL1 (At3g27920) was amplified with MU_pET_GL1_F (5′-AATGGGTCGCGGATCCGAAATGAGAATAAGGAGAAGAGATG-3′) and MU_pET_GL1_R (5′-TGTCGACGGAGCTCGAATTAAGGCAGTACTCAACATCACC-3′). The amplified GL1 CDS fragment was inserted into pSpET using HiFi DNA Assembly Master Mix (NEB, NEB, Ipswich, MA). The pSpET-GL1 was transformed into *Escherichia coli* Rozetta2 (DE3). *E*. *coli* Rosetta2 (DE3) carrying pSpET-GL1 was inoculated into 10 mL of LB-ampicillin and followed by shaking at 37°C overnight. A total of 10 mL of overnight culture was transferred into 100 mL of LB-ampicillin in a 500-mL flask and shaken at 37°C until the OD_600_ reached 0.6. Once the culture reached OD_600_ = 0.6, 1 mM of isopropyl-β-D-thiogalactopyranoside was added, and the culture was further shaken at 20°C for 20 h to complete induction.

Expression and purification of host proteins

The DNA fragments of PAP1 (At1g56650) and GL3 (At5g41315) were cleaved by *Eco*RI and cloned into a shrimp alkaline phosphatase-treated pGEX6P-1 vector using HiFi DNA Assembly Master Mix (NEB, Ipswich, MA) to construct GST-PAP1 and GST-GL3, respectively. Then, each construct was transformed to *E*. *coli* Rosetta2 (DE3). The recombinant proteins were expressed and purified as previously described (Umeyama and Morohashi, 2020). Then, GST-fuzed proteins were purified using glutathione sepharose beads based on the manufacturer’s protocol. Finally, the protein concentration was measured with a Pierce BCA Protein Assay Kit (Thermo Fisher Scientific, Waltham, MA), and 100 µL of the 36.6 µM concentration of proteins was used for further analysis.

Preparation of Hishot protein

The induced culture was collected in a 500-mL bottle followed by centrifugation at 3,500 rpm for 15 min at 4°C After discarding the supernatant, 30 mL of phosphate-buffered saline (PBS) (1,370 mM NaCl, 27 mM KCl, 81 mM Na_2_HPO_4_·12H_2_O, and 14.7 mM KH_2_PO_4_) was added, resuspended, and centrifuged at 3,500 rpm for 15 min at 4°C.

Association of the Hishot complex with the host protein

Lysozyme was added in 10 mL of the bacteria-expressed GL1-Hishot protein to achieve 1 mg/mL as a final concentration. In the case of the RNase or RNase inhibitor treatment, RNase I was added to achieve 10 ng/mL as a final concentration, or RNase inhibitor (SUPERase In; Thermo Fisher Scientific, Waltham, MA) was added to achieve 0.8 U/µL as a final concentration, respectively. Then, the reaction solution was incubated for 30 min at 37°C followed by sonication on ice until the stickiness was gone. The sonicated solution was centrifuged at 9,000 rpm for 15 min at 4°C. Then, 500 µL of the supernatant was transferred into a tube that contained 100 µL of the host protein. Next, 300 µL of a 30% slurry of glutathione sepharose 4B was added in the tube and incubated at 4°C with rotation overnight.

RNA extraction from the Hishot complex and reverse transcription

The reaction extract containing GL1-Hishot proteins and GST proteins was centrifuged at 500 rpm for 10 min at 4°C, and the supernatant was removed. Then, the beads were washed three times with 500 μL of PBS. Next, 350 μL of TRI Reagent (Cosmo Bio, Tokyo, Japan) was added into the beads. The upper layer was transferred into a new tube, and RNase-free DNase I (Sigma-Aldrich, St. Louis, MO) was added to achieve 0.1 U/µL as a final concentration. Then, the RNA was purified by phenol–chloroform extraction followed by ethanol precipitation. The purified RNA was subject to reverse transcription using a ReverTra Ace qPCR RT kit (Toyobo, New York, NY) in a 20-µL reaction volume according to the manufacturer’s protocol.

RT-PCR and qRT-PCR

For semi-qRT-PCR, MU_GL1_seq (5′-GTTCATCACTGCCGCCACAC-3′) and T7 terminator primer (5’-GCTAGTTATTGCTCAGCGG-3’) primer sets were used. After, 25, 30, and 35 cycles of PCR, the reaction was subjected to electrophoresis with 2% agarose gels. For qRT-PCR, the primer sets of YT_GL1_qrt_F (5′-CCGCCACACCTTCTTCTTGTCATC-3′) and YT_GL1_qrt_R (5′-ATACGACGCCGTTAAAGCTCTTGG-3′) were used.
